# Supplementary figures and images for: Xeno-free induced pluripotent stem cell-derived neural progenitor cells for in vivo applications
Source: J Transl Med. 2022 Sep 16;20:421. doi: 10.1186/s12967-022-03610-5 (PMC9482172; doi:10.1186/s12967-022-03610-5)

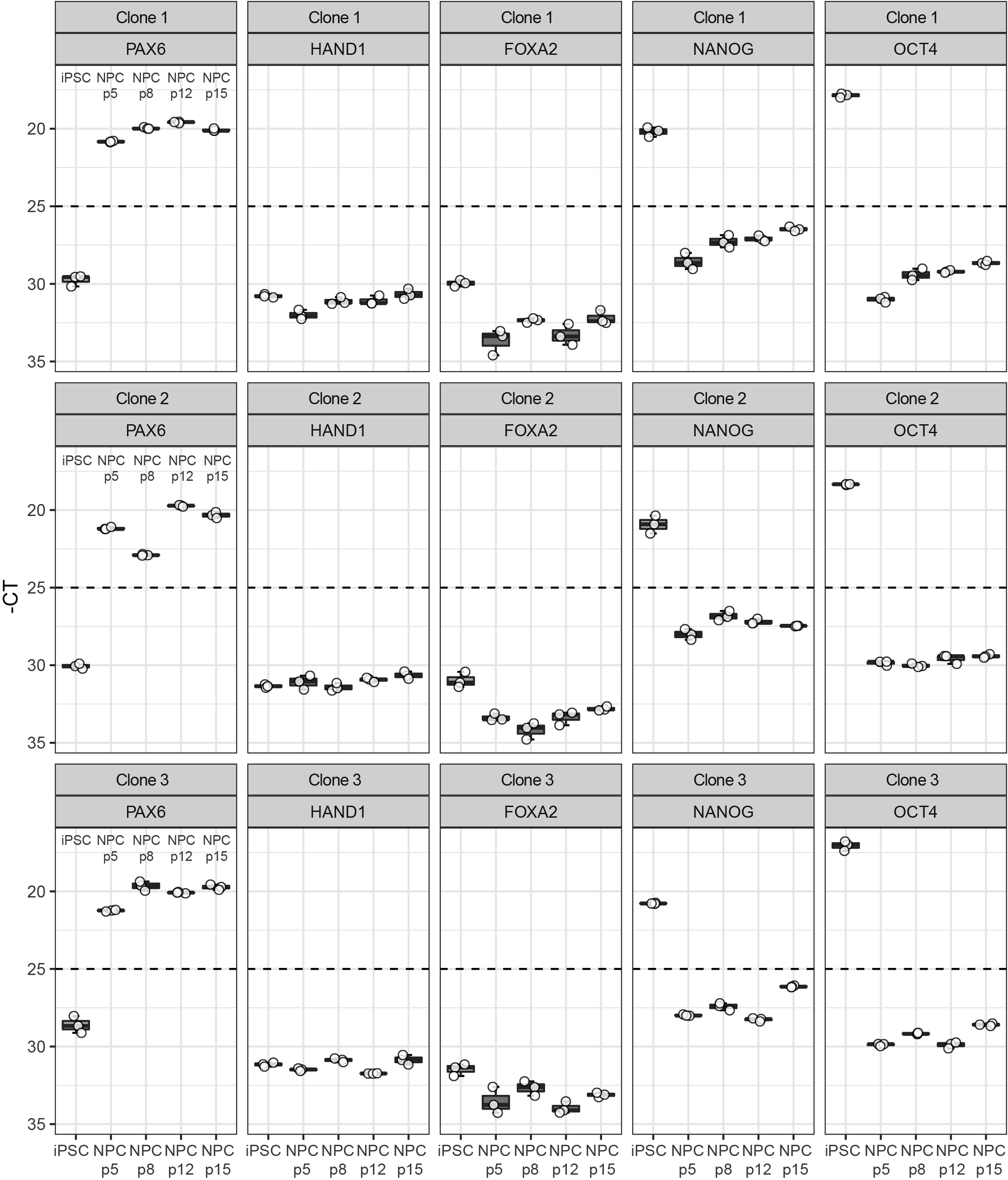

Supplement: Supplementary file 1 — Additional file 1: Figure S1. Expression of different cell lineage markers in NPCs. Gene expression of markers for ectodermal (PAX6), mesodermal (HAND1) and endodermal (FOXA2) cell lineages, as well as for pluripotent cells (NANOG and OCT4), in iPSCs and NPCs from three clonal lines at different passages, measured by qPCR. Boxplots show –CT values. [file 12967_2022_3610_MOESM1_ESM.tif]

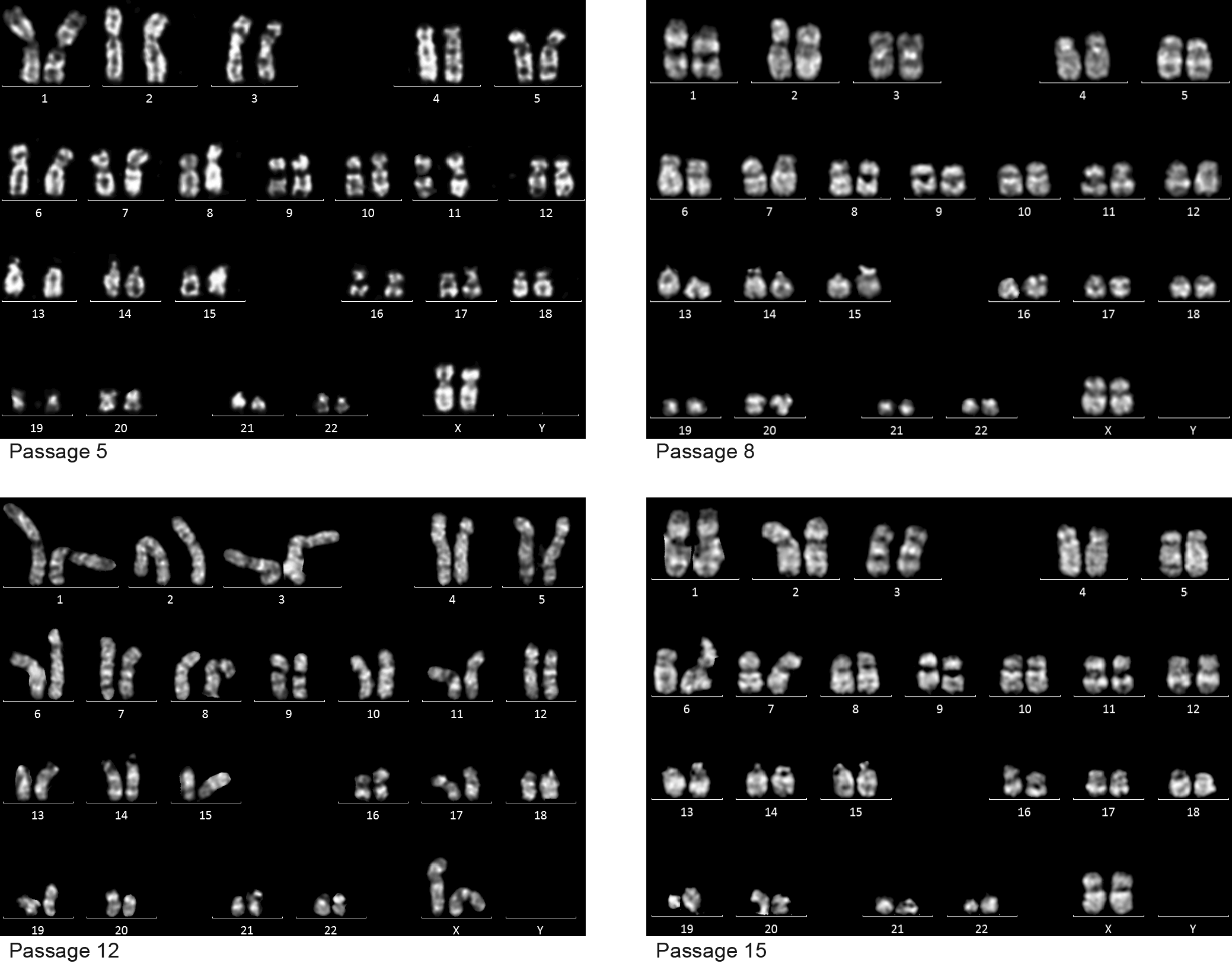

Supplement: Supplementary file 2 — Additional file 2: Figure S2. NPC karyotype. Q-banding karyotype of NPCs at different passages. [file 12967_2022_3610_MOESM2_ESM.tif]

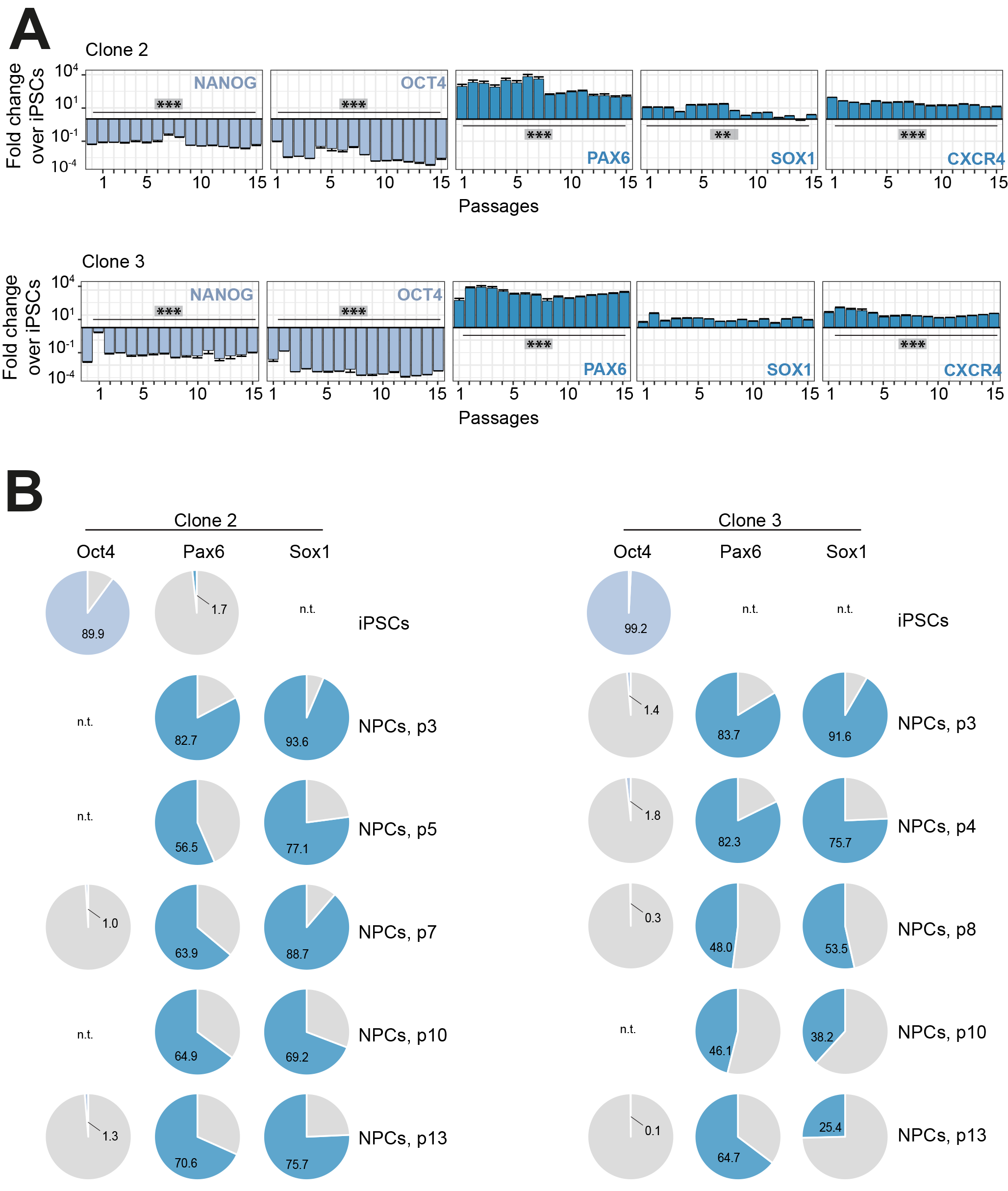

Supplement: Supplementary file 3 — Additional file 3: Figure S3. Characterization of iPSC-derived NPCs from two other iPSC clonal lines. A: Gene expression of pluripotency marker (NANOG and OCT4) and NPC marker (PAX6, SOX1, CXCR4) in NPCs from iPSC clonal lines 2 (upper row) and 3 (lower row) over the course of 15 passages, measured by qPCR. B: Flow cytometry analysis of iPSCs (upper row) and NPCs from iPSC clonal lines 2 and 3 at different passages for Oct4, Pax6 and Sox1. Pie charts illustrate percentage positivity (light/dark blue) for the respective marker and cell type. n.t.: not tested. [file 12967_2022_3610_MOESM3_ESM.tif]

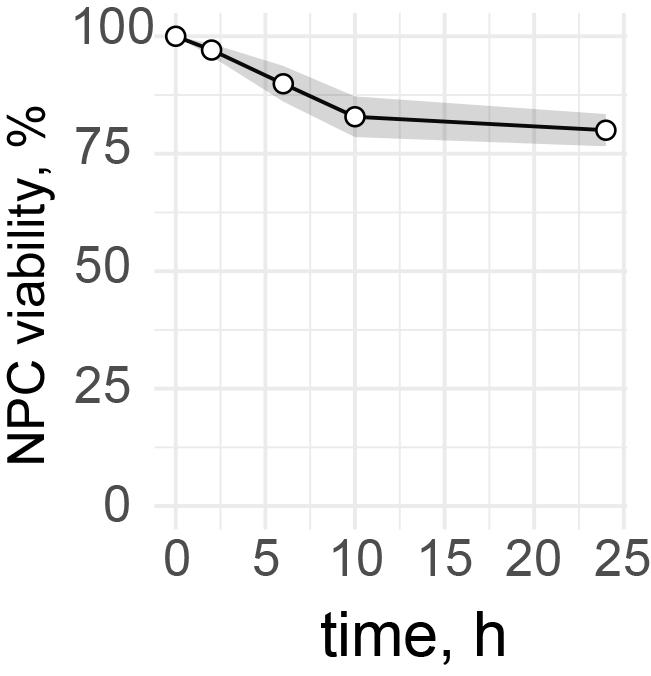

Supplement: Supplementary file 4 — Additional file 4: Figure S4. Cell viability in vitro. Percentage of freshly thawed viable NPCs over the time course of 25h on ice, measured using Vi-Cell XR Cell Viability Analyzer. [file 12967_2022_3610_MOESM4_ESM.tif]

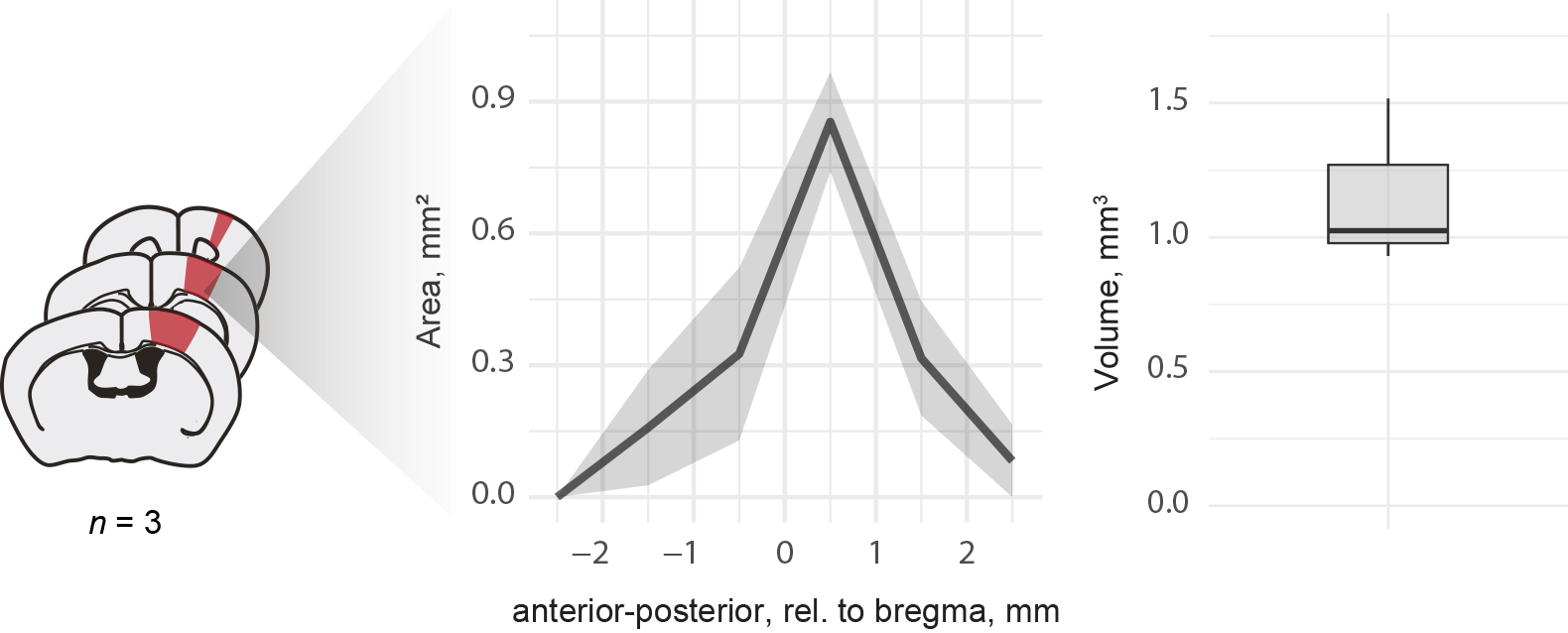

Supplement: Supplementary file 5 — Additional file 5: Figure S5. Lesion size. Quantification of stroke area (plotted as mean ± sem) and stroke volume at 42 dpi. Boxplots indicate the 25% to 75% quartiles of the data. [file 12967_2022_3610_MOESM5_ESM.tif]

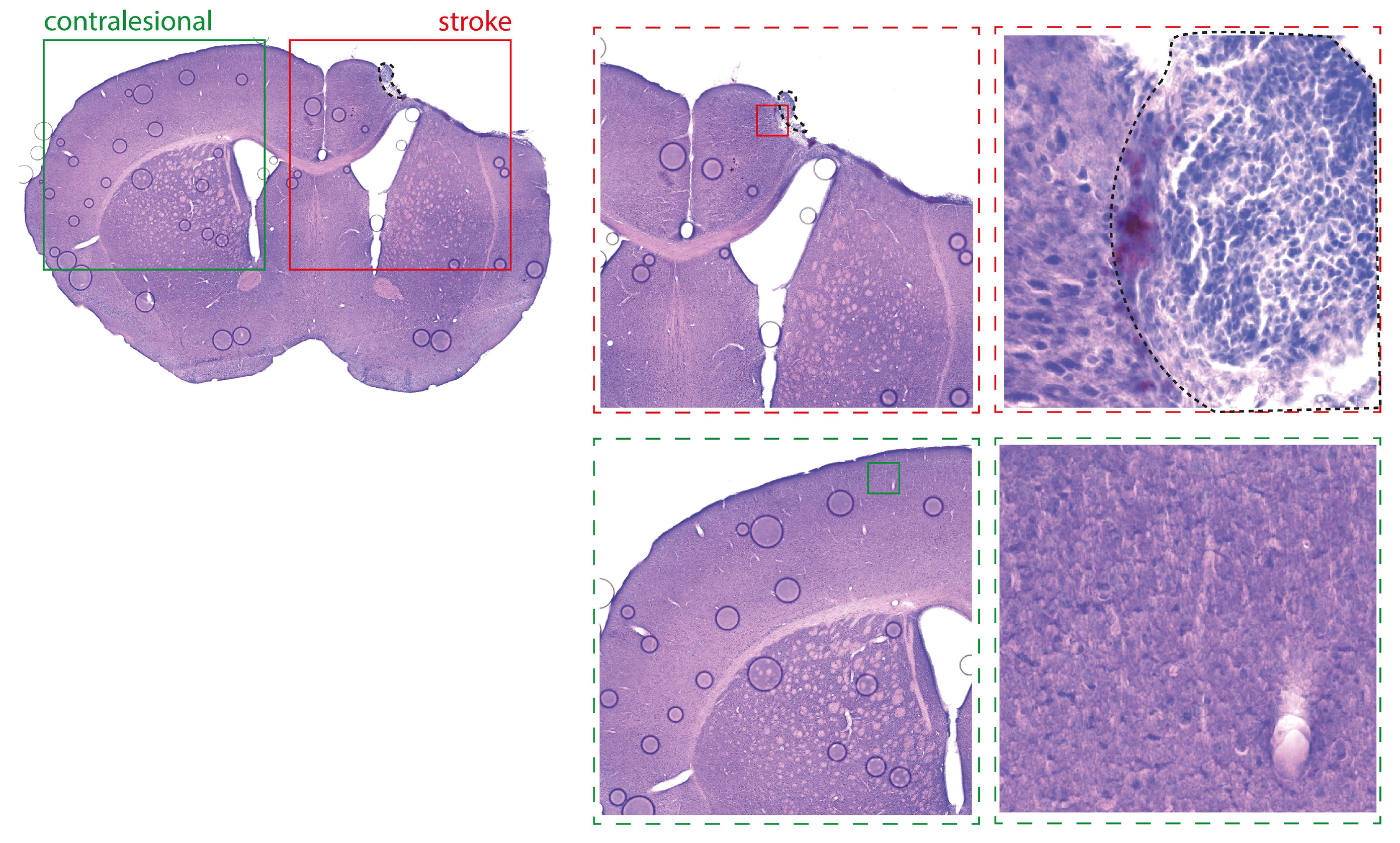

Supplement: Supplementary file 6 — Additional file 6: Figure S6. HE staining. HE staining of the stroked mouse brain, 35d after NPC transplantation. red: stroked hemisphere; green: contralesional, intact hemisphere; black: area of cell transplantation [file 12967_2022_3610_MOESM6_ESM.tif]

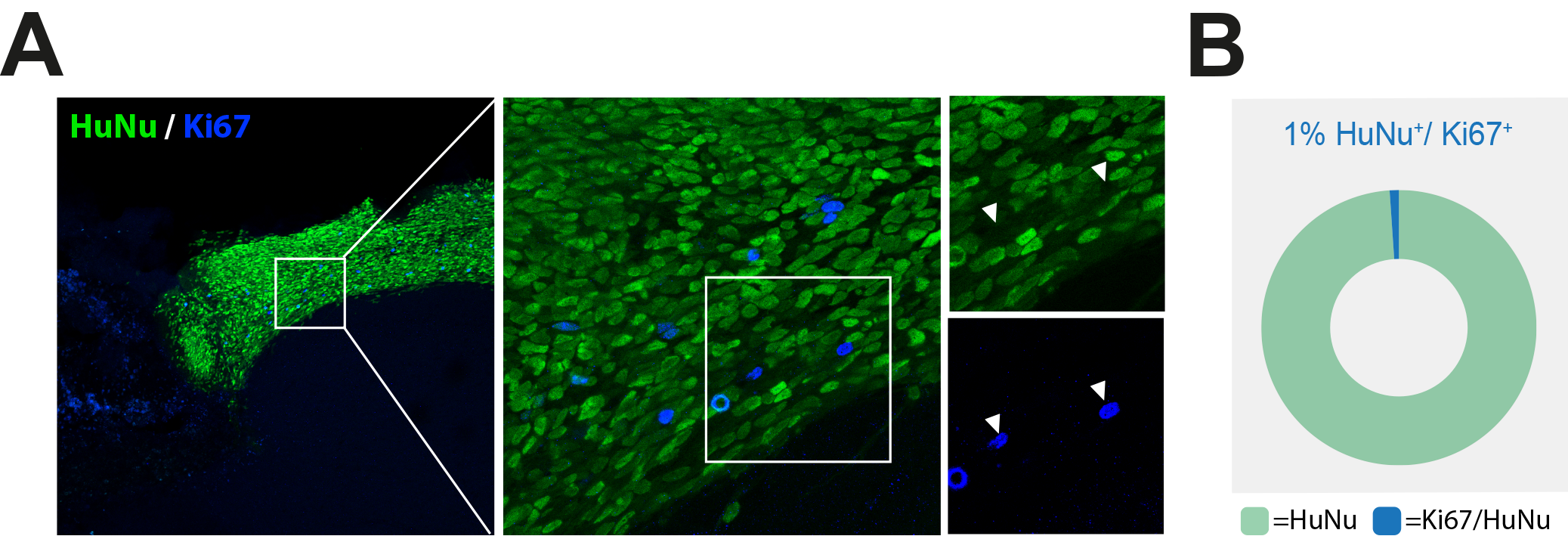

Supplement: Supplementary file 7 — Additional file 7: Figure S7. Quantification of proliferating cells. A: Representative histological staining of transplanted NPCs against proliferation marker Ki67 (blue) and human nuclei (green; grafted cells), 35d post transplantation in stroked Rag2-/- mice. Arrowheads indicate Ki67-expressing cells that are negative for HuNu. B: Quantification of cells positive for HuNu and Ki67. [file 12967_2022_3610_MOESM7_ESM.tif]
