# Supplementary material for: Xeno-free induced pluripotent stem cell-derived neural progenitor cells for in vivo applications
Source: J Transl Med. 2022 Sep 16;20:421. doi: 10.1186/s12967-022-03610-5 (PMC9482172; doi:10.1186/s12967-022-03610-5)
Supplement: Supplementary file 8 — Additional file 8: Table S1. Detailed list of cell culture media and substances. [file 12967_2022_3610_MOESM8_ESM.docx]

**Cell culture media and material**

Neural Induction Medium 1

| DMEM/F12 (50%) 11320-033 | 24ml |
| --- | --- |
| Neurobasal (50%) 21103-049 | 24ml |
| N2- Supplement (100x) | 500ul |
| B27 – Supplement (50x) | 1ml |
| Glutamax (100x) | 500ul |
| hLIF | 10ng/ml |
| CHIR99021 | 4uM |
| SB431542 | 3uM |
| Dorsomorphin | 2uM |
| Compound E | 0.1uM |

Neural Induction Medium 2

| DMEM/F12 (50%) 11320-033 | 24ml |
| --- | --- |
| Neurobasal (50%) 21103-049 | 24ml |
| N2- Supplement (100x) | 500ul |
| B27 – Supplement (50x) | 1ml |
| Glutamax (100x) | 500ul |
| hLif | 10ng/ml |
| CHIR99021 | 4uM |
| SB431542 | 3uM |
| Compound E | 0.1uM |

Neural Stem cell Maintenance Medium (NSMM)

| DMEM/F12 (50%) 11320-033 | 24ml |
| --- | --- |
| Neurobasal (50%) 21103-049 | 24ml |
| N2- Supplement (100x) | 500ul |
| B27 – Supplement (50x) | 1ml |
| Glutamax (100x) | 500ul |
| hLif | 10ng/ml |
| CHIR99021 | 3uM |
| SB431542 | 2uM |
| FGF2 (from passage 2 on) | 5 ng/mL |

Materials

| Cell Adhere Buffer | Stemcell Tech |
| --- | --- |
| Vitronectin XF | Stemcell Tech |
| Accutase Solution | Sigma |
| GlutaMAX-I CTS | Gibco |
| StemMACS™ SB431542 | Miltenyi |
| StemMACS™ CHIR99021 | Miltenyi |
| StemMACS™ Dorsomorphin | Miltenyi |
| N-2 Supplement CTS | Gibco |
| B-27 Supplement XenoFree CTS | Thermo Fisher |
| Animal-Free Recombinant Human LIF | PeproTech |
| StemMACS™ Compound E | Stemcell Tech |
| FGF22 Basic recombinant human protein, Animal-Origin Free | Thermo Fisher |
| Poly-L-ornithine Solution (pLO) | Sigma-Aldrich |
| Laminin-L521 (L-521) | Biolaminin LN |
| Thiazvivin | Sigma-Aldrich |
| DMEM/F12 (50%) | Thermo |
| Neurobasal (50%) | Gibco |
